# Supplementary material for: Splice-Junction-Based Mapping of Alternative Isoforms in the Human Proteome
Source: Cell Rep. Author manuscript; Available in PMC 2020 Jan 15. (PMC6961840; doi:10.1016/j.celrep.2019.11.026)
Supplement: 2 [file NIHMS1546469-supplement-2.zip › DF1/PXD006675/PulmonaryArtery/PulmonaryArtery_6_RYR2_VLDIANVLFHLEQVEHPQR.pdf]

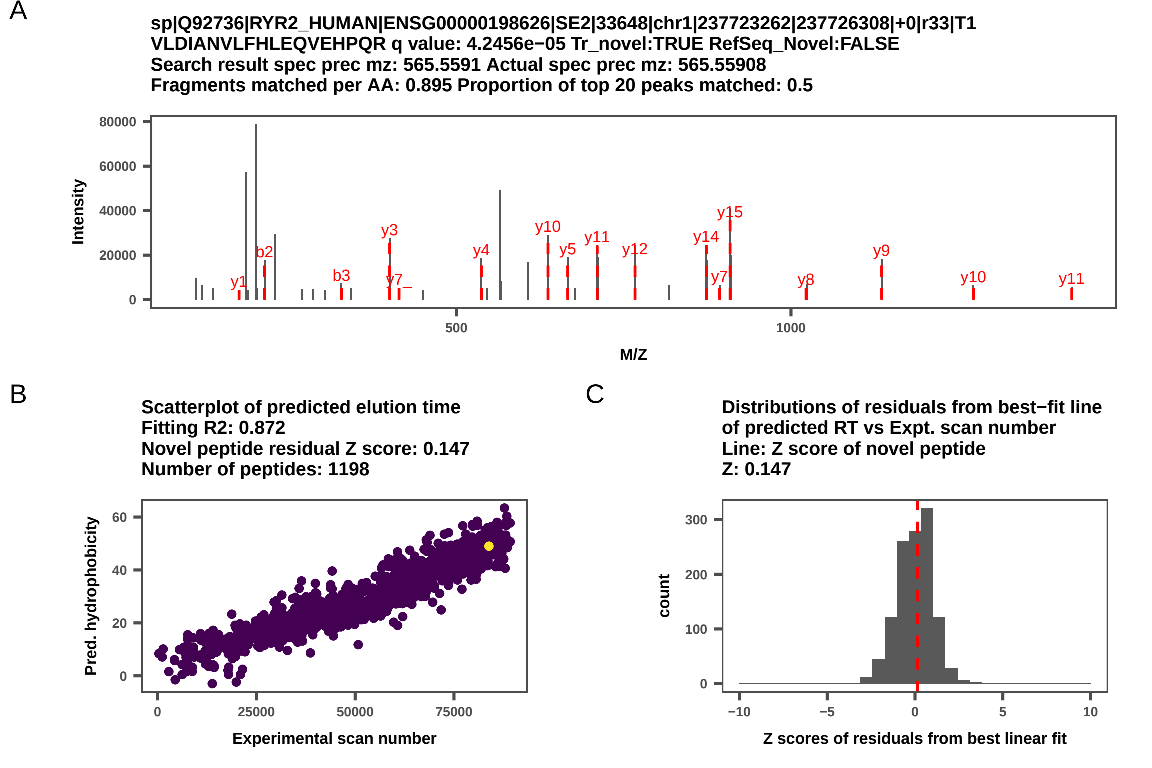
A
sp|Q92736|RYR2_HUMAN|ENSG00000198626|SE2|33648|chr1|237723262|237726308|+0|r33|T1
VLDIANVLFHLEQVEHPQR q value: 4.2456e−05 Tr_novel:TRUE RefSeq_Novel:FALSE
Search result spec prec mz: 565.5591 Actual spec prec mz: 565.55908
Fragments matched per AA: 0.895 Proportion of top 20 peaks matched: 0.5
Intensity
80000
60000
40000
20000
0
y1
b2
b3
y3
y7_
y4
y10
y5
y11
y12
y14
y15
y7
y8
y9
y10
y11
500
1000
M/Z
B
Scatterplot of predicted elution time
Fitting R2: 0.872
Novel peptide residual Z score: 0.147
Number of peptides: 1198
Pred. hydrophobicity
60
40
20
0
0
25000
50000
75000
Experimental scan number
C
Distributions of residuals from best−fit line
of predicted RT vs Expt. scan number
Line: Z score of novel peptide
Z: 0.147
count
300
200
100
0
−10
−5
0
5
10
Z scores of residuals from best linear fit
